# Levosimendan in the emergency department: a useful tool to improve cellular perfusion

**DOI:** 10.1186/cc14233

**Published:** 2015-03-16

**Authors:** G Devia, J Torres, S Lopez

**Affiliations:** 1 Hospital Universitario Mayor, Bogotá, Colombia

## Introduction

Levosimendan is an inotropic seldom used in emergency departments (EDs). It has shown improved mortality in patients with shock [1] with few adverse effects [2]. This work denotes the experience of levosimendan use in the ED of Hospital Universitario Mayor between 2012 and 2014.

## Methods

We present a retrospective study which analyzes the effect, after 24 hours, of levosimendan administration on perfusion parameters of 166 ED patients. Patients had to have shock diagnosis of any cause. Differences between the initial and final mean value of the following parameters were evaluated: lactate, central venous oxygen saturation (ScvO_2_) and venoarterial difference of CO_2_ (DvaCO_2_). Data were stratified according to levosimendan categories (initial or rescue). In addition, association between different variables with mortality was sought. Differences were considered statistically significant at probability levels below 0.05.

## Results

There were no differences in APACHE II values between patients who received levosimendan as initial therapy from those who received it as a rescue measure. A total of 41 patients fulfilled lactate normalization requirements (lactate <2.0 or clearance >50%) (Table 1). Forty-four patients reached normal values of SvcO_2_ and 37 patients of DavCO_2_ after levosimendan initiation. There were no associations between the normalization of lactate, SvO_2_ and DvaCO_2_ and different types of shock. Twenty-nine patients who received initial therapy with levosimendan normalized their lactate values and 12 who received it as a rescue therapy (*P *< 0.05). Sixty-three patients developed hypotension, and none had adverse effects requiring discontinuation of the drug. Hospital mortality was 47.7%. Variables associated with mortality in the study group were lactate value at admission (OR = 1.3, 95% CI = 1.0 to 1.7), the use of vasopressin after start levosimendan (OR = 7.5, 95% CI = 1.9 to 28.6) and the use of norepinephrine before starting (OR = 10.8, 95% CI = 1.9 to 60.7).

**Table 1:** Perfusion variables before and after levosimendan

Variable	Initial	Final
Lactate (mmol/l)	3.3 (0.5 to 18)	2.38 (0.4 to 17)*
SvcO_2_ (%)	56.3 (23 to 85)	65.2 (37 to 91)*
DvaCO_2_ (mmHg)	8.2 (-16 to 26)	7.3 (-2 to 21)

*P < 0.01.McNemar test.

## Conclusion

Levosimendan use in the ED, as initial or rescue therapy, normalizes lactate values and improves the SvcO_2_ after 24 hours, without an increase in adverse effects.

## References

[B1] Landoni G Minerva Anestesiol 2010 76 276 86 20332741

[B2] Mebazaa A JAMA 2007 297 1883 91 10.1001/jama.297.17.1883 17473298

